# Nadroparine-induced skin necrosis on a patient with essential thrombocythaemia: a case report

**DOI:** 10.1186/1757-1626-2-6458

**Published:** 2009-03-10

**Authors:** Manousos-Georgios Pramateftakis, Dimitrios Kanellos, Stefanos Psomas, Ioannis Kanellos

**Affiliations:** 1 Department of Surgery, European Medical Center, Asklipiou 10, 57001 Thessaloniki, Greece

## Abstract

Skin necrosis is a rare but serious complication of subcutaneously administered low-molecular-weight heparin. We report a case of a 53-year-old female patient with skin necrosis induced by subcutaneous administration of nadroparine. The patient suffered from essential thrombocythaemia on a background of chronic myeloproliferative disease. She was admitted to our clinic with a subacute ileus due to endometriosis of the rectosigmoid junction. She underwent a high anterior resection and she received pre- and postoperative antithrombotic prophylaxis with subcutaneous nadroparine on a daily basis. On the 6^th^ and 7^th^ postoperative days, two skin necroses occurred at two injection sites.

## Introduction

Skin necrosis following subcutaneously administered low-molecular-weight heparin (LMWH) is a rare but serious complication. It is believed that skin necrosis, amongst other thrombotic complications observed, are the result of LMWH-induced platelet aggregation followed by intravascular thrombosis. According to current literature, 24 cases of LMWH-induced necroses have been reported. Three of those cases occurred following nadroparine injections. All 24 cases are reporting on patients with thrombocytopenia. Our case is the only thrombocytotic patient amongst all published cases. The aim of this paper is to report on a rare case of skin necrosis following nadroparine injections on a patient with thrombocytosis.

## Case presentation

A 45-year-old Caucasian female patient, with a known history of essential thrombocythaemia on a background of chronic myeloproliferative disease, was admitted to our clinic with a subacute ileus. Laboratory blood tests revealed the following results: Glucose 88 mg/dl, urea 17 mg/dL, creatinine 0.6 mg/dL, SGOT 33 iu/L, SGPT 24 iu/L, γGT 99 iu/L, LDH 418 iu/L, CRP 0.1, total bilirubin 0.7 μmol/L, direct bilirubin 0.35 μmol/L, anti-HCV positive, WCC 9.83 × 10^9^/L, Ht 32.2, PLT 768 (Normal value: >150–300 × 10^9^/L).

All coagulation parameters, including protein S and C activity, antithrombin III activity, activated partial thromboplastin time, prothrombin tine, fibrinogen concentration and fibrinogen degradation products, were within normal range. Computed tomography (CT) of the abdomen (after gastrograffin intake) revealed spleen distention and stenosis at the rectosigmoid junction. The following day the patient was taken to the operation theatre and the patient underwent a high anterior resection, due to endometriosis of the rectosigmoid junction. On postoperative blood test results, the platelet count was constantly high, ranging between 800 & 900 × 10^9^/L. During her stay in our clinic, the patient received piperacillin-tazobactam 4.5 gr intravenously (I.V.) for one day, omeprazole 40 mg I.V. for two days and 0.6 ml of nadroparine subcutaneously to the abdominal wall, on a daily basis. On the sixth postoperative day, the patient complained of pain in the right lower abdomen, at the site of the nadroparine injections and a purple discoloration appeared on the right lower site of the abdominal wall.

**Figure 1:**
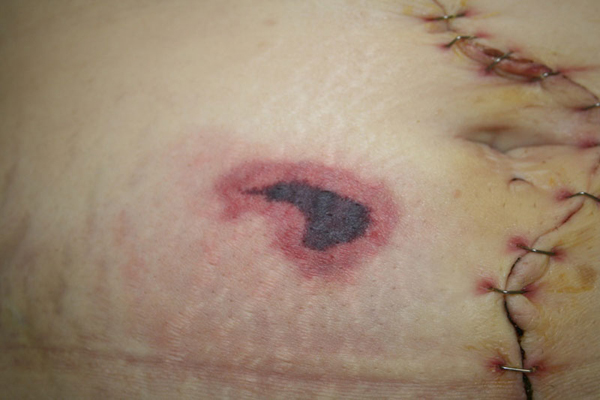
**Necrotic eschar at nadroparine injection site**.

**Figure 2:**
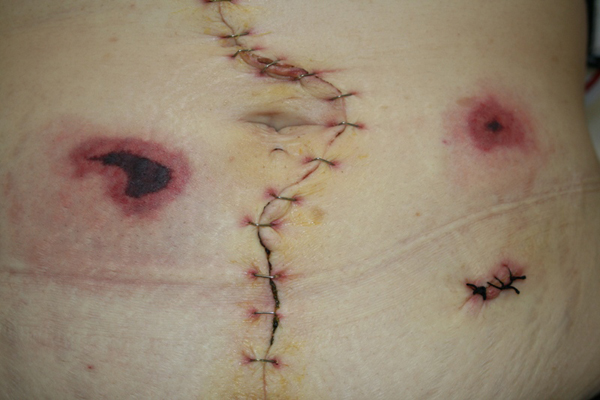
**Second lesion one day later at the left side of the abdomen**.

Within 24 hours of the onset of pain, a necrotic eschar surrounded by erythema had formed on the right lower abdomen (Figure 1). The necrotic margins were well demarcated. One day later a second lesion appeared on the left side (Figure 2). Subcutaneous injections of nadroparine were stopped. The pain at the necrotic areas became severe. The patient developed spiking pyrexia, reaching a maximum of 38.7°C. The fever was thought to be a result of the necroses. The lesions were treated conservatively and they gradually settled. The skin returned to its normal appearance 4-5 weeks later.

## Discussion

Essential thrombocytosis is an acquired myeloproliferative disorder, characterized by the overproduction of platelets by megakaryocytes in the bone marrow in the absence of an alternative cause. Arterial and venous thromboses represent the main risks for patients suffering from essential thrombocytosis [1,2].

Low molecular weight heparins are currently used as the standard anti-thrombotic therapy. Skin necrosis caused by LMWH is a rare complication. 24 cases have been reported up to today, and only 3 of these cases occurred during nadroparine administration [3]. All reported cases occurred on patients with concomitant thrombocytopenia (platelet count <300,000 cells/ml) rather than thrombocytosis, as was the case in our patient. In our case, the determination of Platelet Factor 4 (PF4)-induced antibodies tested positive on Enzyme-Linked ImmunoSorbent Assay (ELISA). Even though our patient had an increased number of platelets, an absolute or even relative decrease of the platelet count is often the only abnormality observed during routine laboratory tests in patients with LMWH-induced skin necroses.

Heparin-dependent IgG antibodies, which activate platelets via their Fc receptors, are regularly found on patients with LMWH-induced skin necroses, independent of the type of LMWH used or of whether the necroses occur locally at the injection sites or further away [4,5]. These antibodies are specific for complexes consisting of heparin and PF4, which are recognized by the antibody, resulting in antibody-mediated vascular injury [6,7]. It is believed that heparin causes platelet aggregation in vessels surrounding the site of injection, with subsequent thrombosis. The presence of fibrin thrombi in the veins and capillaries of fresh histopathological specimens lends further strength to this proposition. Histological examination of LMWH-induced skin necrosis specimens reveals microvascular thrombi in the dermal vessels with minimal inflammation and without signs of vasculitis [3].

According to current literature, skin necroses occur most of the times between the 6^th^ and 12^th^ day after the first injection [8]. It is possible that LMWH has a low immunogenic profile and therefore requires repeated exposure in order to lead to skin necrosis [3]. The diagnosis of heparin-induced platelet aggregation should be strongly suspected whenever necrosis occurs at an injection site. Once skin necrosis is noticed or suspected, the initial therapeutic action is to immediately stop the LMWH therapy. The administration of any other type of (fractioned or unfractioned) LMWH is not recommended, due to a well known cross reaction of the heparin-dependent antibodies amongst the above products. Should further anticoagulation be necessary, one has the option of acetyl-salicylic acid (ASA) or protamin [7,1,9]-[11]. Even though Coumarins may be used, there is a published report on coumarin-induced skin necrosis following HIT syndrome [12].

## Conclusion

In conclusion, skin necrosis following subcutaneously administered low-molecular-weight heparin (LMWH) is a rare but serious complication. It represents a localized manifestation of heparin-induced platelet aggregation and thrombosis.

## List of abbreviations

LMWH: Low molecular weight heparin; SGOT: Serum Glutamic Oxaloacetic transaminase; SGPT: Serum Glutamic Pyruvic Transaminase; γGT: Gamma-glutamyl Transferase; LDH: Lactate Dehydrogenase; CRP: C-Reactive Protein; WCC: White Cell Count; HCV: Hepatitis C Virus; Ht: Hematocrit; PLT: Platelet Count; PF4: Platelet Factor 4; ASA: Acetylsalicylic Acid; HIT: Heparin-induced thrombocytopenia; Fc: Fragment, crystallizable.

## Consent

Written informed consent was obtained from the patient for publication of this case report and accompanying images. A copy of the written consent is available for review by the Editor-in-Chief of this journal.

## Competing interests

The author(s) declare that they have no competing interests.

## Authors' contribution

MGP, SP and IK performed the patient's surgery. MGP, DK, SP and IK assessed the patient on a daily basis and observed the skin abnormalities. MGP and SP wrote the manuscript and performed the data analysis. DK performed the data collection, the image editing, the manuscript drafting and the revision. IK analyzed and interpreted the patient data and was a major contributor in revising the manuscript and giving the final approval. All authors read and approved the final manuscript.

## References

[B1] Elliott MA Tefferi A Thrombosis and haemorrhage in polycythaemia vera and essential thrombocythaemia Br J Haematol 2005 128 275 290 10.1111/j.1365-2141.2004.05277.x 15667529

[B2] Michiels JJ Berneman Z Bockstaele DV van der Planken M De Raeve H Schroyens W Clinical and laboratory features, pathology of platelet-mediated thrombosis and bleeding complications and the molecular etiology of essential thrombocythemia and polycythemia vera: therapeutic implications Semin Thromb Hemost 2006 32 174 207 10.1055/s-2006-939431 16673274

[B3] Handschin AE Trentz O Kock HJ Wanner GA Low molecular weight heparin-induced skin necrosis - a systematic review Langenbecks Archives of Surgery 2005 390 249 254 10.1007/s00423-004-0522-7 15570433

[B4] Tietge UJF Schmidt HH-J Jackel E Trautwein C Manns MP Low molecular weight heparin-induced skin necrosis occurring distant from injection sites and without thrombocytopenia Journal of Internal Medicine 1998 243 313 315 10.1046/j.1365-2796.1998.00304.x 9627146

[B5] Santamaria A Romani J Souto JC Lopez A Mateo J Fontcuberta J Skin necrosis at the injection site induced by low-molecular-weight heparin: case report and review Dermatology 1998 196 264 265 10.1159/000017889 9568422

[B6] Eika C Inhibition of Thrombin Induced Aggregation of Human Platelets by Heparin Scand J Haematol 1971 8 216 5093999 10.1111/j.1600-0609.1971.tb01976.x

[B7] Eika C On the Mechanism of Platelet Aggregation Induced by Heparin, Protamine and Polybrene Scand J Haematol 1972 9 248 4626630 10.1111/j.1600-0609.1972.tb00937.x

[B8] Ojeda E Perez MC Mataix R Arbelo R Jimenez S Campo C Balda I Skin necrosis with a low molecular weight heparin Br J Haematol 1992 82 620 10.1111/j.1365-2141.1992.tb06477.x 1336672

[B9] Landolfi R Di Gennaro L Novarese L Patrono C Aspirin for the control of platelet activation and prevention of thrombosis in essential thrombocythemia and polycythemia vera: current insights and rationale for future studies Semin Thromb Hemost 2006 32 251 259 10.1055/s-2006-939436 16673279

[B10] Van Genderen PJJ Mulder PGH Weleboer M Van de Moesdijk D Michiels JJ Prevention and treatment of thrombotic complications in essential thrombocythaemia: efficacy and safety of aspirin Br J Haematol 1997 97 179 184 10.1046/j.1365-2141.1997.d01-2127.x 9136963

[B11] Griesshammer M Bangerter M van Vliet HH Michiels JJ Aspirin in essential thrombocythemia: status quo and quo vadis Semin Thromb Hemost 1997 23 371 377 10.1055/s-2007-996111 9263354

[B12] Faraci PA Deterling RA Stein AM Warfarin Induced Skin Necrosis Surg Gynecol Obstet 1978 146 695 644427

